# The limits of personalization in precision medicine: Polygenic risk scores and racial categorization in a precision breast cancer screening trial

**DOI:** 10.1371/journal.pone.0258571

**Published:** 2021-10-29

**Authors:** Jennifer Elyse James, Leslie Riddle, Barbara Ann Koenig, Galen Joseph

**Affiliations:** 1 Institute for Health and Aging, University of California, San Francisco, California, United States of America; 2 Department of Humanities and Social Sciences, University of California, San Francisco, California, United States of America; Hong Kong Genome Institute, HONG KONG

## Abstract

Population-based genomic screening is at the forefront of a new approach to disease prevention. Yet the lack of diversity in genome wide association studies and ongoing debates about the appropriate use of racial and ethnic categories in genomics raise key questions about the translation of genomic knowledge into clinical practice. This article reports on an ethnographic study of a large pragmatic clinical trial of breast cancer screening called WISDOM (Women Informed to Screen Depending On Measures of Risk). Our ethnography illuminates the challenges of using race or ethnicity as a risk factor in the implementation of precision breast cancer risk assessment. Our analysis provides critical insights into how categories of race, ethnicity and ancestry are being deployed in the production of genomic knowledge and medical practice, and key challenges in the development and implementation of novel Polygenic Risk Scores in the research and clinical applications of this emerging science. Specifically, we show how the conflation of social and biological categories of difference can influence risk prediction for individuals who exist at the boundaries of these categories, affecting the perceptions and practices of scientists, clinicians, and research participants themselves. Our research highlights the potential harms of practicing genomic medicine using under-theorized and ambiguous categories of race, ethnicity, and ancestry, particularly in an adaptive, pragmatic trial where research findings are applied in the clinic as they emerge. We contribute to the expanding literature on categories of difference in post-genomic science by closely examining the implementation of a large breast cancer screening study that aims to personalize breast cancer risk using both common and rare genomic markers.

## Introduction

The long-held promise of genomic medicine is to “transform screening and diagnostic testing,” making it more precise and personalized to each individual [1–4]. Yet, two decades after the mapping of the human genome, the translation of emerging genomic findings into prevention, clinical care, and public health remains a daunting challenge. Since the completion of the first draft sequence of the human genome, thousands of genome wide association studies (GWAS) have been performed in an effort to identify single nucleotide polymorphisms (SNPs) that may contribute to disease risk or be protective against disease [5]. Unlike rare mutations in high-penetrance susceptibility genes like BRCA1/2 that convey substantial increases in disease risk for a small number of people, each SNP typically has a modest disease association, but in combination may explain variation in disease incidence in the general population [6].

### Polygenic risk scores

Scientists are now calculating these cumulative effects of SNPs by generating polygenic risk scores (PRS) which may be used, together with traditional risk factors, to stratify individuals into risk categories for diseases such as cancer, coronary artery disease (CAD) and psychiatric disorders [7]. These polygenic risk scores constitute the latest frontier of genomic medicine, garnering huge investments in the basic science of disease risk that are expected to yield significant advances in clinical care. While research on polygenic risk scores has been ongoing for the past decade, its clinical validity is still debated, with evidence varying by disease [8–10]. As Yanes and colleagues argued in a recent review, “There are several methodological and reporting elements that need to be addressed before clinical utility and implementation can be fully realized” [7]. Only recently have practices for developing and implementing PRS begun to be standardized [7,11,12]. Nevertheless, commercial genomic sequencing laboratories are offering an array of both clinical and direct-to-consumer tests that include PRS as part of their “holistic” risk prediction products for a variety of diseases and conditions [13–15].

Another fundamental concern about polygenic risk scores is their reliance on repositories of SNPs that do not represent the full range of human genetic variation, raising the question: If clinical validity is established, for whom will it be valid [16]? Allele frequencies (or the incidence of a genetic variant in a population) vary by genetic ancestry, yet GWAS have predominantly included participants of European descent [17]. Although the proportion of individuals of non-European ancestry in GWAS databases has increased in recent years, individuals of European ancestry are still estimated to compose 78% of all GWAS [18]. Samples from individuals of African or Hispanic/Latin American ancestry only increased by 2.5% and approximately 0.5%, respectively, between 2009 and 2016; in the case of indigenous peoples (including Native Americans, Australian Aboriginals and Pacific Islanders), representation decreased slightly during that period [17]. As a result, polygenic risk scores may be “fundamentally less informative in populations more diverged from the GWAS study cohorts” [16,19]. Breast cancer is among the diseases with the most PRS research underway, but how PRS informs risk prediction remains inconclusive, in part due to the homogeneity of GWAS [20]. The lack of representative GWAS has been recognized as a key obstacle in the project of Precision Medicine. Initiatives like the All of Us study [21,22], the Clinical Sequencing Evidence-Generating Research Consortium (CSER) [23], the Human Genome Reference Program (HGRP) [24], the PRS Diversity Consortium [25], and others [26–28], have been designed to address both the underlying science and clinical translation, as well as longstanding debates about the role of race in medicine, genetics, and genomics.

### Race and genomics

The extensive social science literature on race and genomics demonstrates how scientists, despite intentions otherwise, reify race as a biological category [29–32]. Yet, in the post-genomic era, some scientists have argued that the use of race and ethnicity is an appropriate proxy for genetic ancestry that will help us to understand and reduce persistent health disparities [33–37]. Given that the burden of disease incidence and mortality is often unequal across racial and ethnic groups, using genetics and genomics to understand racial differences in health outcomes can be “framed as an anodyne ethical obligation” [38]. However, doing so reifies notions of biological difference using socially defined categories. As Catherine Bliss argues, “a collective concept of race that presumes that there are, or were at some point in the past, discreet genetic groups that have tracked along continental lines and that those differences are the fundamental basis for our folk and political groupings of white, black, Asian, Native American, and Pacific Islander is a fallacy that will always lead to social inequality” [39].

The lack of consensus—and transparency—about how race and ethnicity are defined and used in genomics research exacerbates the challenge of how to operationalize difference in genomic science and medicine [40]. Self-identified race/ethnicity categories are used to recruit and track research participants in order to fulfill reporting requirements to the NIH and other funding institutions [41,42]. Yet, the use of self-identified race often moves beyond a “*descriptive* mode” to “produce biological *attribution*” [43]. Self-identified categories are thus imbued with a biological meaning, reified by the constant search for difference along racial and ethnic demarcations of difference. Recently, the use of “geographic ancestry” or “biogeographic ancestry” in place of self-reported race/ethnicity have been suggested as ways to identify genuine biological variation tied to population variability across geographic regions rather than based on social categories of race and ethnicity. However, the potential for slippage between ancestral geographic variation and racial categories based in social hierarchies is ever-present [29].

In this article, we detail how these issues play out in one specific effort to translate genomic medicine into practice: a pragmatic clinical trial that aims to personalize breast cancer screening using PRS in combination with traditional risk factors such as age and family history. Our ethnography of the trial provides timely, critical insights into how categories of race, ethnicity and ancestry are being deployed in the production of genomic knowledge and in medical practice. Specifically, we show how the conflation of social and biological categories of difference can shape the understandings and practices of scientists, clinicians and research participants/patients. Our research highlights the potential harms of practicing genomic medicine using under-theorized and ambiguous categories of race, ethnicity, and ancestry.

### The WISDOM trial: A case study of precision breast cancer screening

Annual mammography beginning at age 40 has been the standard of care in the United States since the 1980s. However, over the past 20 years, significant controversy and debate about the best approach to breast cancer screening has emerged and persisted [44]. In 2009, the US Preventive Services Task Force introduced changes to its breast cancer screening guidelines, recommending that annual mammography for all women aged 40–74 be replaced by biennial screening, and that screening in the 40s should be individualized by taking patient context into account, including the patient’s values regarding specific benefits and harms (USPSTF 2009). In 2015, the American Cancer Society updated its prior guideline that all women over 40 screen annually, recommending that women 40–44 have the opportunity to begin annual screening; that women 45–54 screen annually; and that women 55 and older transition to biennial screening or have the opportunity to continue screening annually [45]. While the American College of Radiology continues to argue that annual mammography starting at age 40 should not be changed [46], some epidemiologists and clinicians maintain that annual mammography results in too many false positives and unnecessary treatments, and that a more targeted approach could result in less over-diagnosis of low risk breast cancers like ductal carcinoma in situ (DCIS) without an increase in lethal cancers [47].

The WISDOM trial (Women Informed to Screen Depending on Measures of Risk) (ClinicalTrial.gov: NCT02620852) aims to inform and resolve these intense policy debates by comparing comprehensive risk-based screening (RBS) to annual mammography in a randomized controlled trial [44,48]. WISDOM’s risk-based screening approach, which incorporates genomic risk assessment, is intended to move away from the “one-size-fits-all” method by differentiating between individuals at high, average and low risk of breast cancer and recommending screening schedules accordingly. The WISDOM trial is designed to test the hypothesis that risk-based screening will be as safe, less morbid and have greater healthcare value than annual mammography [48]. WISDOM is the first effort in the U.S. to recommend breast cancer screening according to a risk assessment that incorproates individual genomic risk rather than only population characteristics. The trial aims to recruit 100,000 participants aged 40–74 and was originally funded by PCORI, the Patient Centered Outcomes Research Institute, and subsequently by the National Cancer Institute and private funders.

**Fig 1:**
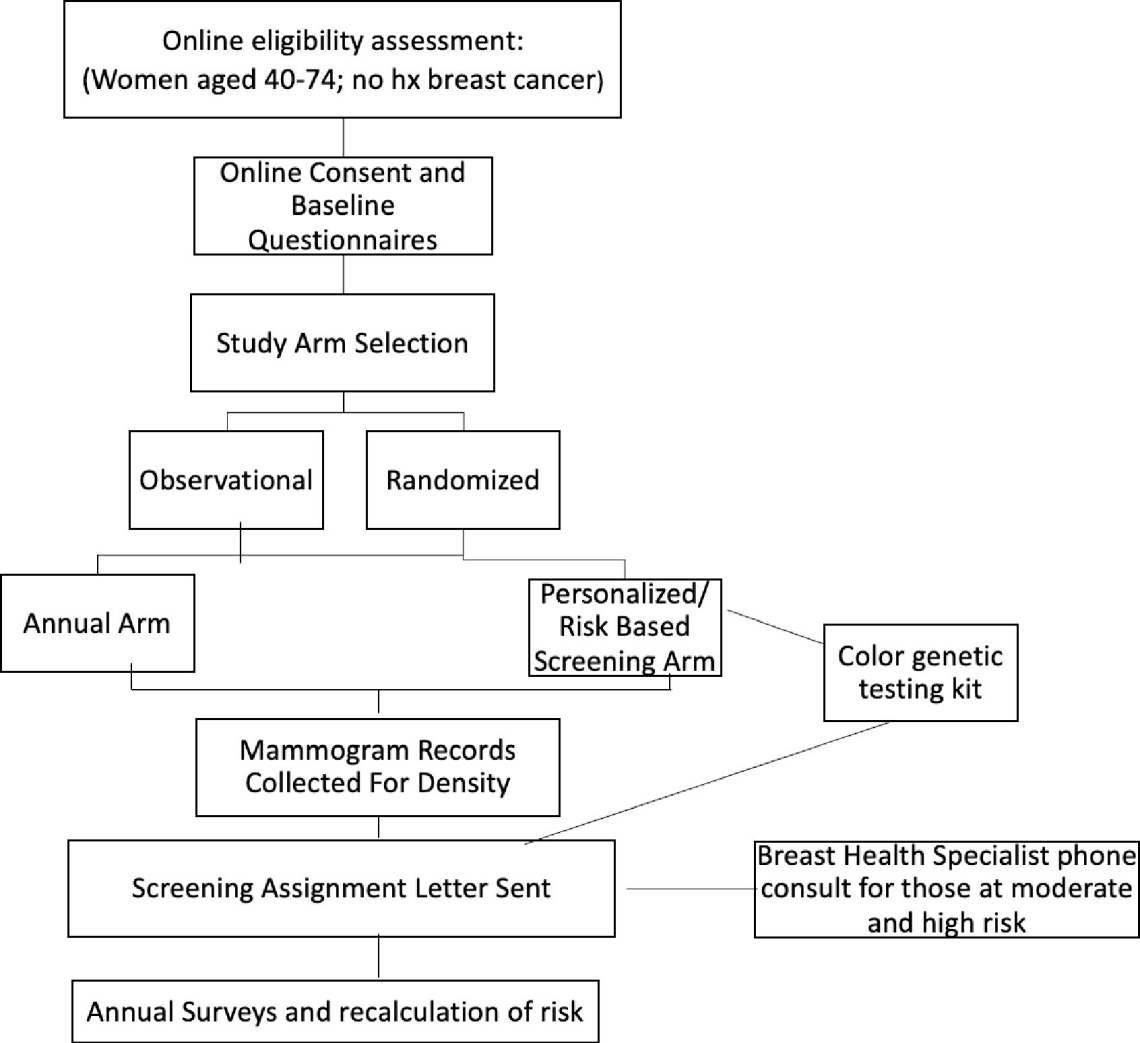
WISDOM trial flow diagram.

As a pragmatic clinical trial, WISDOM is structured in a number of innovative ways, with the goal of incorporating new data as it emerges so that results remain relevant at the end of the study and can immediately be translated into clinical practice. WISDOM is designed to be both adaptive and pragmatic, meaning that in contrast to standard clinical trials, it aims to bridge research and clinical practice by testing new scientific knowledge in real-world settings [49]. Also, to reflect real world circumstances, WISDOM is “preference tolerant” which enables participants to decide whether to be randomized into a specific study arm, or if they have a strong preference, to choose their study arm: annual mammography or RBS (Fig 1). Finally, as a pragmatic trial, WISDOM is attempting to prove clinical validity and clinical utility simultaneously; WISDOM not only tracks the validity of its risk prediction model, but if and how the model, including the evolving Polygenic Risk Scores and associated screening recommendations, is taken up by participants to guide real-world screening decision making and clinical behavior.

Participants in both RBS and annual arms are evaluated for breast cancer risk using the Breast Cancer Surveillance Consortium (BCSC) risk calculator which incorporates age, self-reported race, family history (first degree relative with breast cancer), breast density and breast biopsy history [48,50]. Those in the RBS arm also receive genomic screening for nine high and moderate penetrance breast cancer genes (ATM, BRCA1, BRCA2, CDH1, CHEK2, PALB2, PTEN, STK11, and TP53), and >200 single nucleotide polymorphisms (SNPs) associated with breast cancer risk.

The SNPs are used to calculate a Polygenic Risk Score (PRS), which is then combined with the BCSC score to produce a five-year breast cancer risk estimate. While there is no universally accepted method for calculating PRS, “In general, PRSs are constructed by summing the total number of risk alleles an individual carries, weighted by an estimation of the impact of each allele on disease risk” [7] (p.166). Participants in the RBS arm are assigned, via electronic letter, to one of five screening schedules according to their combined BCSC and PRS, with thresholds for these screening schedules set by the study investigators. Participants may receive one of the following recommendations: 1) delay screening (for average risk participants below age 50); 2) discontinue routine screening (for low risk participants over age 70); 3) have a mammogram every two years (for participants age 50–74 at average risk or age 40–49 with risk ≥ 1.3%); 4) have annual mammograms (for those at moderate risk); or, 5) have annual mammograms *and* annual MRI (for those at highest risk). All of these screening recommendations fall within at least one current U.S. breast cancer screening guideline (e.g., USPSTF, ACS). However, these screening recommendations are just that: recommendations. Participants are expected to confer with their primary care providers about their screening schedule, and any screening or prevention services must be paid for by the participant or her insurance company [48].

### Categorization of race in the WISDOM trial

**Fig 2:**
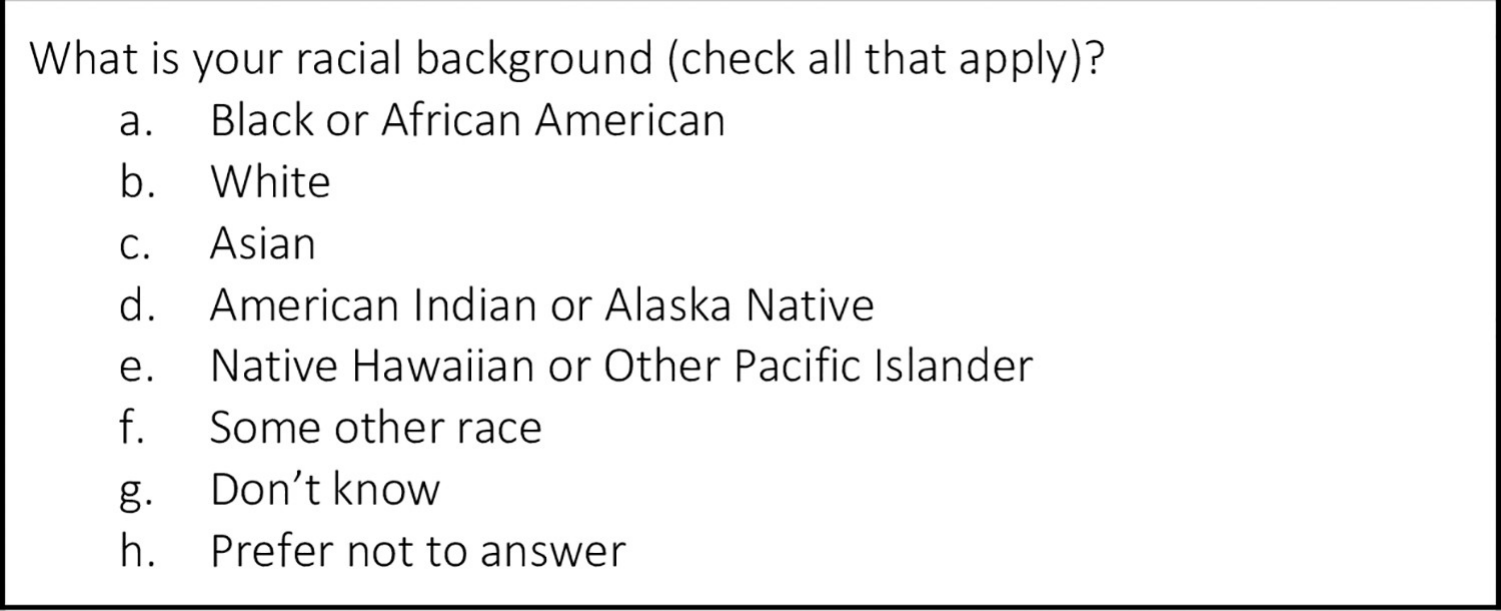
Racial background questions in the WISDOM trial.

WISDOM uses multiple survey questions to ascertain self-reported racial and ethnic identity. These questions reflect the U.S. Office of Management and Budget (OMB) categories required by PCORI, NIH and other research funding agencies, but go beyond the required data to collect more granular categories of identity, similar to the U.S. census (Fig 2).

**Fig 3:**
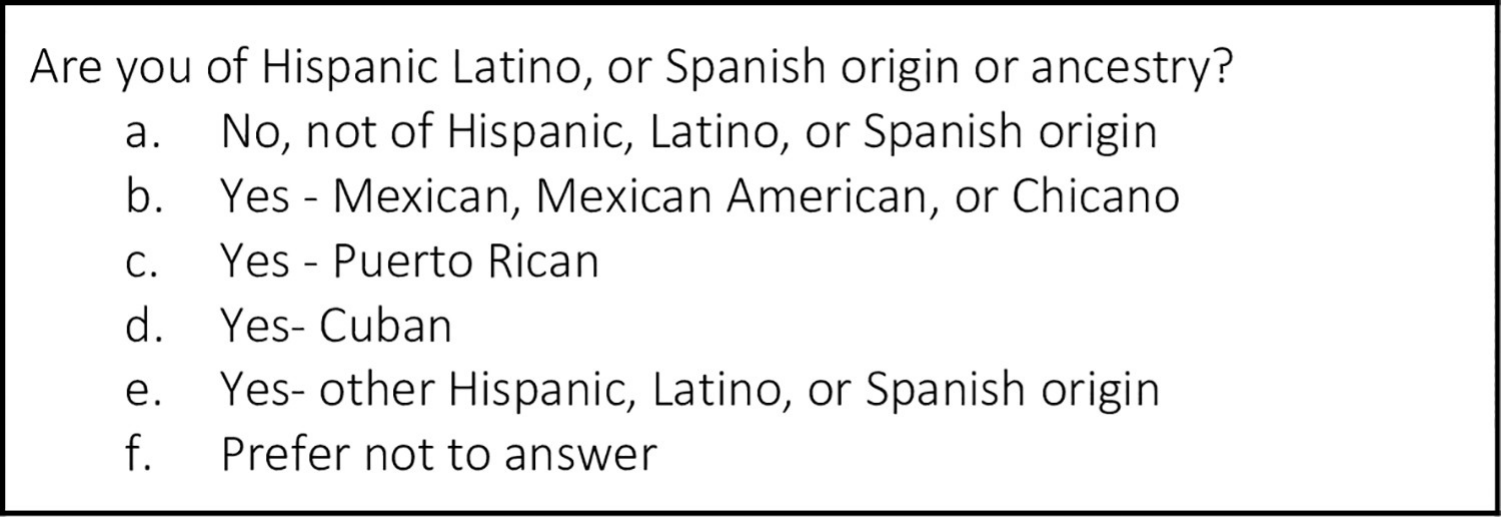
Hispanic ancestry questions in the WISDOM trial.

Participants are asked to provide more detailed origin information if they have selected Asian or Native Hawaiian or Other Pacific Islander, although this information is not used in the risk calculation. All participants are then asked, “Are you of Hispanic, Latino or Spanish origin or ancestry?” (Fig 3), yet WISDOM classifies all these subcategories as “Hispanic.”

**Fig 4:**
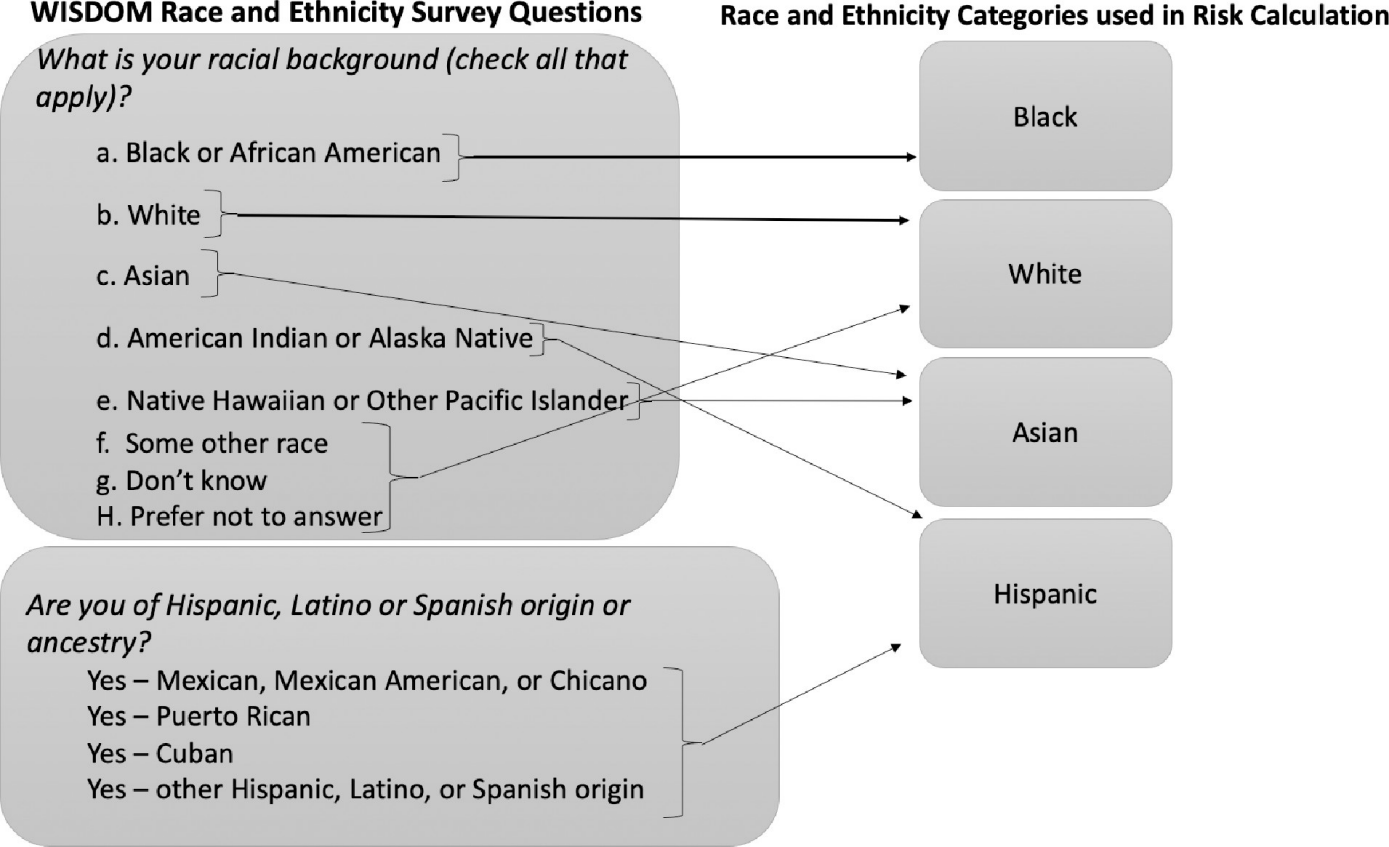
WISDOM race and ethnicity encoding.

The WISDOM trial uses this race/ethnicity information in two ways: to calculate the BCSC score and the PRS. The BCSC risk calculator is calibrated (i.e., assessed for agreement between predicted probability and observed risk) for non-Hispanic white, non-Hispanic black, Asian/Native Hawaiian/Pacific Islander, Native American/Native Alaskan, and Hispanic women. The data (and risk estimates) for non-Hispanic white women is used if the race/ethnicity is “other,” “unknown,” or if a participant selects more than one race. This is because white women have the highest rates of breast cancer in most age groups. Thus, BCSC assigns the most conservative risk estimate in these cases. In the context of the WISDOM trial, the BCSC risk estimate is calculated slightly differently; these categories are collapsed and participants are assigned to one of four “race codes”: Black, white, Asian and Hispanic (Fig 4). These are the same four categories used to calculate the PRS. Much has been written about the limitations of utilizing race in clinical risk assessment, including critique of the BCSC model [51,52]. While it is important to understand the implications of racial categorization and race correction in the BCSC calculator and how it is used in conjunction with PRS in the WISDOM trial, we focus in this paper on racial categorization in PRS due to its novelty and the potential down-stream effects of reifying the idea of genetic racial differences. WISDOM is operating within exisiting clinical structures and using the best-available tools for calculating traditional breast cancer risk. In this paper, we offer insight into this novel calculation of genomic risk to help shape the debate around this practice before its full integration into clinical care.

As described above, the PRS is calculated based on a large panel of SNPs. The number of SNPs used varies based on an individual’s self-identified race. In addition to a base set of SNPs used for all participants, there are “ethnic-specific SNPs” that have been shown to be predictive of or protective against breast cancer in certain groups [53]. Additionally, allele frequencies and odds ratios are used to give different weights to specific SNPs depending on the frequency of occurrence and strength of prediction in various racial and ethnic groups, thus offering a more targeted PRS based on whether an individual has been categorized as Black, white, Asian or Hispanic.

While the WISDOM survey questions define the category of “Hispanic” as broadly inclusive, the research underlying the allele frequencies and ethnic-specific SNPs used in the WISDOM PRS define “Hispanic or Latino” as a population with Indigenous, European and African ancestry admixture. Thus, the race and ethnic identity categories from which participants may select on the WISDOM surveys and the race and ethnicity categories WISDOM uses to calculate their breast cancer risk are distinct.

At the start of the trial, WISDOM, following norms for the application of PRS, implemented a PRS that was only calibrated to participants of European ancestry (meaning the score was based on SNP data collected from largely European GWAS and therefore used European-derived allele frequencies). WISDOM investigators have updated the PRS as new GWAS studies were published, as permitted in an adaptive trial design [49], specifically to improve the calibration for other ancestral groups [48,54].

## Methods: Ethnography of the WISDOM trial

Recognizing the potential of the WISDOM trial to influence breast cancer screening policy and the science of precision medicine, we embarked upon an ethnographic and empirical bioethics study of the WISDOM trial [55–57]. Over the past five years, our team of ethnographers has observed thousands of meetings of more than 20 ongoing WISDOM trial working groups, which meet regularly to address and implement various aspects of the study (e.g., risk thresholds for screening recommendations, statistical methods, return of genomic results). In fieldnotes, we have documented how study procedures were developed and have evolved; how genomic screening was implemented; how decisions were made; and the experiences of variously positioned participants, staff, and investigators in the process. We also conducted 31 key informant interviews with WISDOM staff, clinicians and investigators; study collaborators such as patient advocates, payors and policy makers; and experts working in academic and commercial genomics.

To elucidate the experience of receiving genomic risk information and personalized screening recommendations in the context of the trial, we audio recorded 57 telephone results-disclosure sessions in which WISDOM participants learned that they were at elevated risk for breast cancer. We then conducted semi-structured interviews with these participants at two time points (2 weeks and 6 months post-results disclosure) to explore how they understood their risk and made screening decisions in light of the information provided to them in the trial. In addition, we conducted 22 semi-structured interviews with participants designated as low and average risk, as well as with 4 primary care providers and 5 WISDOM Breast Health Specialists who deliver results by phone to high and moderate risk participants.

Recordings of interviews and results disclosure sessions were transcribed and analyzed using standard techniques based on a modified grounded theory approach [58]. We used a qualitative analysis software that enables searching and retrieval of coded text and audio (Atlas.ti). Our research team worked collectively to code initial transcripts and develop a codebook. Subsequently, each transcript was coded by one member of the team and then reviewed by another to ensure consistent application of the codes; discrepancies were resolved through discussion and consensus. Transcripts and coded data were then discussed with the full research team to explore emerging themes and interpret the data.

Drawing on the full methods described here, this paper focuses on a case study of one participant whose risk estimate and screening recommendation shifted substantially when race/ethnic specific PRSs were implemented. This participant’s racial/ethnic self-identification and the trial’s racial/ethnic classification conflicted in such a way as to shed light on the fluidity and subjectivity of the categories themselves. We use this case to illustrate the larger themes we have observed in our data, and to demonstrate the challenges of a cutting-edge genomic trial relying on antiquated racial and ethnic categories to produce complex risk assessments.

### Findings: Re/Mis-categorizing race

**Fig 5:**
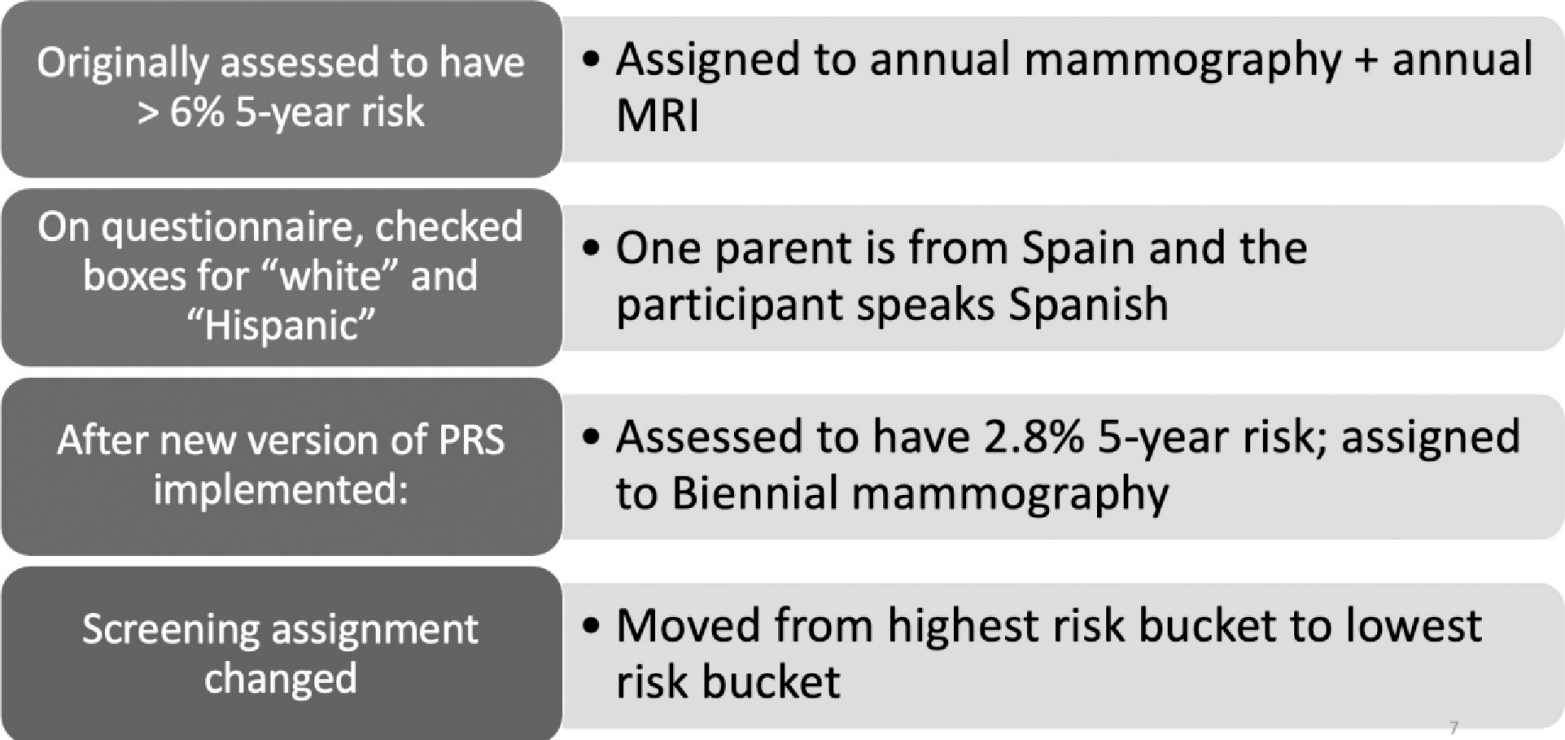
WISDOM participant Joanna.

Approximately two years into the trial, WISDOM implemented a new polygenic risk score with newly validated SNPs intended to be more accurate for women of African, Asian or “Latin-American” ancestry [53]. At that point, approximately thirteen thousand participants had joined the study, with a much smaller number having received genetic test results due to randomization and the option to choose a study arm. Further, not all in the personalized arm had completed their genetic testing. Nevertheless, the new PRS algorithm changed the risk estimate for many participants in the trial. For 35 of them, the new algorithm lowered their risk estimate substantially enough to change their screening recommendation. While screening recommendations are updated each year to reflect changes reported in the annual questionnaires (such as age or a new breast cancer diagnosis in the participant’s family), these 35 women were informed of their new screening assignments off schedule to ensure timely receipt of their updated risk assessment and corresponding screening recommendation. One participant who fell into this group was “Joanna” (Fig 5).

Joanna was a 62-year-old woman who, when she enrolled in WISDOM, identified herself as white and Hispanic. After selecting her race as white and answering “yes” to the question “Are you of Hispanic, Latino or Spanish origin or ancestry?” she indicated that she was “other Hispanic.” Joanna had originally been given the recommendation to have an annual mammogram and an annual MRI, the most aggressive screening schedule in the WISDOM trial (referred to as “Q6”). Despite not having traditional risk factors such as a family history of breast cancer or a BRCA mutation, Joanna’s combined BCSC risk estimate and PRS indicated that she had a greater than 6% five-year risk for breast cancer, a threshold established by WISDOM investigators based on the approximate five-year risk for a woman with a BRCA2 mutation. After the new race/ethnicity calibrated version of the PRS went into effect, Joanna’s assessed risk dropped dramatically, from 6.09% to 2.8% five-year risk, moving her from the most aggressive to the least aggressive screening recommendation for her age: biennial mammography. Soon after, Joanna received a call from a WISDOM Breast Health Specialist, a genetic counselor, to explain the change:

“So one of the things that changed is that we added some new genetic markers that really are much better equipped to account for people who have ancestries that aren’t Caucasian. So for individuals who have either mixed ancestries, such as yourself, or who were of ancestries other than Caucasian ancestry, our calculator–you know, we weren’t sure how good of a job it was doing. So, we added these things that can make it more accurate for people who just have different ancestries.”

Joanna responded by saying, “Okay. So now you don’t consider Spanish or from Spain Caucasian? Spaniards are not considered Caucasian?” The Breast Health Specialist (whom, it should be noted, does not have a role in determining the risk thresholds or classifying race and ethnicity and is only tasked with relaying this information to participants) responded:

“This is all a little bit of a moving target. People from Spain are often considered Hispanic, but are also considered Caucasian. And when we look at things from a genetic and ancestry perspective, it can be a little bit muddled. So it’s difficult when we’re–you know, a research study has to draw lines in the sand and the computer has only so much programming it can take and, you know, there are limitations to how granular we can get with some of these things. So a lot of times the calculator will run as, quote/unquote, Hispanic if a person has Spanish ancestry and clicks that they are Hispanic, the same as somebody from Latin America. But really, ancestrally it’s different. So it’s a little bit complicated.”

When we interviewed Joanna and asked about the change in screening recommendation she responded, “I didn’t quite get it. So I was just happy that the percentage had gone down.” When asked about her self-identified race and ethnicity and her understanding of how WISDOM had used that information, she described herself as “more European” and said, “I don’t know if there was confusion because I’m really of European heritage, not Hispanic as in South America or Central America." In response to a question about her thinking when she completed the WISDOM questionnaires she noted, “when I see the word Hispanic, I think well, because you know, I speak Spanish and my mom lives in Spain, but after I researched it a little bit more, it doesn’t seem like it’s the same.” She described asking the Breast Health Specialist about this saying, “since ancestry, ethnic ancestry was a factor then I told her, you know, I don’t know if my study was accurate because I’m from Spain—and I thought maybe they had confused—because I think I had looked up Hispanic and it seems like Hispanic is South America.” She felt like the Breast Health Specialist didn’t really know the answer to these questions and said she didn’t ask for more explanations. Despite being left with some confusion and limited understanding of how the trial had interpreted her race/ethnicity, Joanna reiterated her faith in the trial and even her willingness to share information about the trial with her friends.

At the time Joanna received her original screening recommendation, she took it to her primary care provider (PCP) at her Health Maintenance Organization (HMO), who said the HMO would not cover the cost of the recommended MRI. Initially, when she believed her risk to be elevated, Joanna had planned to pay for the MRI out of pocket, but, she told us, she just hadn’t “gotten around to it” yet. After she received the revised screening recommendation, Joanna told us she planned to continue getting annual mammograms as she had done before joining the WISDOM trial, rather than switching to biennial mammograms as WISDOM recommended. She also noted she might still try to get an MRI as additional surveillance, “just in case.” The ideal scenario in the context of this pragmatic trial would have been for Joanna to take what she learned in WISDOM into the clinic, first sharing the information with her PCP and then following the study’s screening recommendation. Yet, she was first inhibited from following the high-risk screening recommendation by her HMO, and was then disinclined to follow the revised low-risk screening recommendation due to her own concern about her risk. WISDOM’s intital assessment of her as high risk seemed to have contributed to her concern.

Joanna’s case points to some of the critical challenges and complexities of implementing precision medicine using current categories of race, ethnicity, and ancestry. First, it illustrates the disjuncture between self-reported race/ethnicity and geographic ancestry. While WISDOM uses self-reported race and ethnicity for its calculation of the PRS, the allele frequencies in reference databases used to determine risk are specific and may not represent the range of ancestries among the WISDOM participants. For example, the allele frequencies used in WISDOM’s Hispanic/Latina PRS is based on the 1000 Genomes Project database [59]. While the 1000 Genomes Project represents the most diverse dataset currently available, it remains quite limited. The Hispanic/Latino populations are represented by four groups: individuals of Mexican Ancestry from Los Angeles, USA; Puerto Ricans from Puerto Rico; Colombians from Medellin, Colombia; and Peruvians from Lima, Peru [59]. The populations in the database are discordant with both WISDOM’s and the OMB definition of Hispanic/Latino, which includes people from Spain. While the 1000 Genomes Database includes individuals from Spain in its European population, Latin American populations are recognized as having ancestral “admixture” of European, African and Indigenous populations at varying proportions. The boundaries between these populations are not clearly defined. The calibration of WISDOM’s PRS for Hispanic/Latino accounts for indigenous variation in the Americas, including variants found in indigenous populations that are protective against breast cancer and thus lower breast cancer risk estimations for Hispanic/Latino women compared to white women [60]. While Joanna accurately answered the questions about her origins, WISDOM’s self-identified race and ethnicity questions do not map on to the ancestral assumptions of the polygenic risk score. It is not that WISDOM’s classification of Joanna was “incorrect.” Rather, these categories are arbitrary and don’t account for the heterogeneity that exists within categories of race, ethnicity, language and country of origin.

In a prescient interview, one of our key informants, a breast cancer genetics researcher not affiliated with WISDOM, insisted that WISDOM must be using biological ancestry markers derived from saliva samples, telling us, “They must be. Because… they must be, they are using genome-wide array…I would be surprised that they would use self-reported ancestry to modify anything about the risk.” The researcher went on to talk specifically about the ancestral variability of people who identify as Hispanic, saying, “There’s [sic] a lot of people from Latin America who are… second or third generation European, so they have no indigenous American ancestry. And so you could never–I would never adapt a genetics risk score based on self-reported ethnicity. I would never do that.” In her view, these social categories of race and ethnicity may not correlate with geographic ancestry and thus may be inaccurate for determining an individual’s risk profile.

WISDOM investigators have recognized the challenges of utilizing self-identified race in the genomic context. A group of WISDOM investigators and clinicians meet bi-weekly as a Risk Thresholds Committee to review issues related to the risk algorithm and the cutoff points and triggers for the various screening recommendations. In the fourth year of the trial, investigators spent several Risk Thresholds Committee meetings discussing race and ethnicity, including debating whether to move from self-reported race/ethnicity to ancestral markers for calculating PRS. There were some technical concerns: the trial does not receive ancestry information from Color, the lab sequencing WISDOM participants’ DNA. Additionally, according to one WISDOM investigator, “the use of ancestry in a more continuous way has not really been developed or validated for PRS.” Most PRS, regardless of whether genetic ancestry or self-identified race is used, rely on categorical variables (e.g. Black, White, Asian, Hispanic/Latino), irrespective of the complexity of racial identification and the fluidity between the categories. WISDOM’s implementation of PRS mirrors the state of the field. What makes the WISDOM context different is that risk information is being returned to patients, which has, in some cases, highlighted the disconnect between (potentially complex/multiple category) self-identification and the categories WISDOM employs in its PRS analysis. The challenges of mapping self-identified race onto ancestral variation exist across the field; *however*, *unlike WISDOM*, *in most PRS research the results are not returned to participants in a way that makes the disconnect apparent or with the expectation that participants use the data for their clinical care*. The WISDOM investigators are exploring the possibility of using ancestry to calibrate PRS, but this will require a substantial investment of funding and research and may still be several years away.

While Joanna questioned why her Spanish origin was considered Hispanic, she had the benefit of discussing her risk with a Breast Health Specialist and ultimately left the conversation feeling comfortable with her risk assessment, although she planned to follow a different screening schedule than the one WISDOM ultimately recommended. The majority of participants receive a screening recommendation letter without the accompanying conversation (Breast Health Specialist consults are only actively offered to participants with moderate or high risk, though any participant may request a consultation). These participants may assume that the “race” listed on their screening assignment letter, which is based in the collapsed risk assessment categories, is a mistake. For example, several participants who identify as Filipina have reported on their questionnaires that they are of “Spanish origin” and received a letter listing their ethnicity as “Hispanic.” They have written emails to the WISDOM study noting that they do not identify as Hispanic, and that this categorization does not feel appropriate. The trial investigators are currently discussing changing the letters to reflect the participant’s self-identified race rather than the “race code” used in the risk calculation. While this may be less confusing for participants, it raises new questions about transparency and the implications of the trial listing the participant’s self-identified race in a letter conveying risk information that is based on a set of collapsed race categories, which may be substantially different.

Shifting to the use of ancestral markers might result in more accurate risk assessments for participants in the WISDOM trial who are not of European or solely European ancestry, yet this move to ancestry will not solve the problem of race in genomics. Geographic ancestry may be a novel way to draw distinctions between groups, but slippage between race, ethnicity, and ancestry is inevitable and may just as easily reify race as biological and further legitimate risk assessment based on these categorizations. While ancestry may feel less political than race, it is not value neutral. Scientists are still making decisions about where to draw boundaries and how to create categories of difference. Further, ancestry—like all genetics—is contingent on the reference genomes. As reference genomes become more diverse, the interpretation of an individual ancestral admixture can change. Thus, like racial categories, ancestry is somewhat contingent on a variety of factors shaped by social and political forces. As such, it may be viewed as simply the most recent incarnation of race-based medicine.

## Discussion

As Bowker and Star have shown in their exemplary work on classification, *Sorting Things Out*, “race operates via an Aristotelian binary system of mutual exclusivities (i.e., white or black, but not both, ‘you either are, or you are not’) and a more abstract system of prototypical thinking. With regard to the latter, racial norms emerge from a range of socially resonant possibilities and practices that are co-produced amidst heterogeneous beliefs and cultural associations” [61] (pp. 61–63). The WISDOM trial brings cutting-edge science from bench to bedside, in order to assess the real-world implications of applying genomic knowledge to cancer screening. Yet, the tools used to categorize differences in risk in medicine–social classifications of self-identified race and ethnicity—are imprecise and outdated. Across science and medicine, we suffer from a lack of definitional clarity when it comes to race and ethnicity [62]. Categories of race, ethnicity, origin, and ancestry are often used interchangeably or without a clear definition or explanation of how they were ascertained. Race can be used as a proxy variable for genetic ancestry, epidemiological trends, or the product of racism, all of which play different roles in health and illness.

The WISDOM trial illuminates the challenges of using race as a “risk factor” in the implementation of “precision” breast cancer screening. Numerous researchers have offered cautions about the continued use of race as a biological category in medicine, including in breast cancer risk assessment [51,52,63]. WISDOM illustrates the difficulty of applying race-based algorithms to the breast screening context where there are potential harms of both too little or too much screening. As a pragmatic trial, WISDOM blurs the line between research and clinical care by asking participants to take what they have learned in a research setting and, working with their health care providers, apply those findings to their clinical care. Thus, questions about the potential harms of racialized risk construction are not academic; risk assessments and screening recommendations are delivered to WISDOM participants with the expectation that they will be implemented in the clinic. WISDOM minimizes this risk by only recommending guideline-adherent screening regimens, but it applies them based on experimental assessments of risk that may have different implications for some categories of participants. If a participant joins the WISDOM study and is mixed race, with one parent who is of Asian descent and one parent who is of African descent, this individual will receive a different risk score depending on whether she marks herself as Asian, Black, or both. While this may not matter in the clinical context, where a patient is working in collaboration with her provider and using this score as just one tool to aid shared decision-making, it may have different implications in a clinical trial like WISDOM, where participants are unlikely to be aware of how their endorsement of certain racial and ethical categories on a survey is being used in their risk assessment and correlated screening recommendation. Further, if the WISDOM model of risk-based screening is adopted as standard clinical practice, one must consider the possibility that a patient could be precluded from accessing screening, or be subject to the harms of over-screening, due to a risk score that is produced based on self-identified—or possibly practitioner-identified—race and ethnicity. The purpose and implications of the classification must be clear to both those making the classification and those being classified.

The issues we raise here are not intended to critique the WISDOM trial’s practices, but rather to raise questions about how to equitably implement “precision population health.” Our ethnographic work reveals how the troubled relationship of race and genomics continues in present-day clinical applications of genomic science. If we move our lens away from individual patient care and take a population health perspective, the issue of the alignment of self-reported race/ethnicity and genetic ancestry is less consequential. Ancestry and self-reported race often match and the majority of people in WISDOM don’t see their risk shift dramatically due to PRS [64,65]. But when we are considering individual women receiving clinical care based on this score, providing an “inaccurate” PRS raises ethical concerns. The example of the WISDOM trial highlights the complications and challenges of implementing precision medicine on a population scale in a country as ancestrally diverse as the U.S.

As we write this paper, WISDOM is considering implementing low pass whole genome sequencing to facilitate access to a broader range of SNP data which could potentially identify more or better race/ethnic specific SNPs. The investigators are also considering redesigning their race and ethnicity questionnaires to allow participants to self-identify with more granularity. At this time however, it is unknown if or how either intervention would allow for improved risk prediction or how either may reinforce the “molecularization” of race [38]. Further, both directions may exacerbate a focus on race as a defining difference between people, masking differences that exist both between and within ancestry groups. Sequencing the human genome established that people are more than 99% alike genetically, yet scientists have remained captivated by the less than 1% of the genome that varies from person to person, with a particular focus on the fraction of that variation that is associated with geographic ancestry groups [42]. In the WISDOM study alone, participants have selected more than 90 unique combinations of self-reported race and ethnicity. These have in turn been mapped onto only four categories for risk assessment. Many WISDOM investigators acknowledge this process is flawed. The current approach in the field of precision medicine is to continue to refine the categories we use to define race and ancestry. We argue instead for the ultimate goal of identifying risk assessment tools that can be applied equitably across socially determined racial and ethnic groups, and genomically determined ancestral groups. If this goal is not possible, we are perpetuating a system of separate but (un)equal medicine.

Further, the WISDOM trial demonstrates that these problems will not be solved simply by recruiting a diverse study population, an oft-proposed remedy. Simply conducting more research on race and genomics will not solve these critical questions. Expanding GWAS databases to include more diverse populations or shifting away from race to a focus on ancestry in medicine will not solve these challenges either. Semantic change alone will not solve the problem of race in medicine; ancestry is not a neutral scientific concept, devoid of the political repercussions of race and ethnicity. Ancestry may be merely the current incarnation of race-based medicine, allowing scientists to create groups and reify biological difference. We must critically examine the intersection of the racialization of medicine and the project of precision medicine. Will race-specific genomics open new possibilities for the treatment and prevention of disease or further exacerbate existing health disparities? What is clear today is that we are still far from true “precision” screening. Personalized medicine remains dependent on imperfect methods of categorizing race and ethnicity, based in largely European ancestry GWAS. Trials attempting to influence clinical care must consider these limitations and, as a field, we must consider the ethical implications of diverse enrollment in genomic research.

The stark disparities in breast cancer mortality are an indicator that work must be done to better understand how to prevent, screen for, and treat breast cancer. However, by continuing to focus efforts on identifying genetic differences, we may miss opportunities to understand and intervene upon the systemic racism that contributes to disparate health care and health outcomes. Alondra Nelson critically reminds us that genetics can be both “friend and foe”; scientists imbue meaning onto genetic markers which “in and of themselves have no meaning or value” [66] (p. 18). The historical use of race in medicine is complex and rife with examples of abuse, misuse and the marginalization of people of color. Many scholars have examined the possibility that these harms will be exacerbated in the post-genomic era [67]. Our ethnography of the WISDOM trial provides critical insights into how categories of race, ethnicity and ancestry are currently being deployed in the production of genomic knowledge and medical practice, along with concomitant harms and benefits and their potentially uneven distribution across populations.
